# Development of the choroid plexus and blood-CSF barrier

**DOI:** 10.3389/fnins.2015.00032

**Published:** 2015-03-03

**Authors:** Shane A. Liddelow

**Affiliations:** ^1^Department of Neurobiology, Stanford UniversityCA, USA; ^2^Department of Pharmacology and Therapeutics, The University of MelbourneParkville, VIC, Australia

**Keywords:** choroid plexus, development, blood-CSF barrier, cerebrospinal fluid, brain patterning, epithelia, neuroependyma

## Abstract

Well-known as one of the main sources of cerebrospinal fluid (CSF), the choroid plexuses have been, and still remain, a relatively understudied tissue in neuroscience. The choroid plexus and CSF (along with the blood-brain barrier proper) are recognized to provide a robust protective effort for the brain: a physical barrier to impede entrance of toxic metabolites to the brain; a “biochemical” barrier that facilitates removal of moieties that circumvent this physical barrier; and buoyant physical protection by CSF itself. In addition, the choroid plexus-CSF system has been shown to be integral for normal brain development, central nervous system (CNS) homeostasis, and repair after disease and trauma. It has been suggested to provide a stem-cell like repository for neuronal and astrocyte glial cell progenitors. By far, the most widely recognized choroid plexus role is as the site of the blood-CSF barrier, controller of the internal CNS microenvironment. Mechanisms involved combine structural diffusion restraint from tight junctions between plexus epithelial cells (physical barrier) and specific exchange mechanisms across the interface (enzymatic barrier). The current hypothesis states that early in development this interface is functional and more specific than in the adult, with differences historically termed as “immaturity” actually correctly reflecting developmental specialization. The advanced knowledge of the choroid plexus-CSF system proves itself imperative to understand a range of neurological diseases, from those caused by plexus or CSF drainage dysfunction (e.g., hydrocephalus) to more complicated late-stage diseases (e.g., Alzheimer's) and failure of CNS regeneration. This review will focus on choroid plexus development, outlining how early specializations may be exploited clinically.

## Introduction

The first description of the lateral ventricular choroid plexuses is credited to Herophilos (c. 335–280 B.C.) by Galen of Pergamon (see Liddelow, 2011)—he named the structure the “chorioid mennix,” “chorioid” being taken from the outer vascular plexus of the fetus. Two centuries years later, Rufus of Ephesus (c. 100 A.D.) suggested the term “chorioid tunic” be used to describe both the ependyma and choroid plexus. A century and a half of silence followed which was broken when Vesalius reported the gross anatomy of the choroid plexus of the lateral ventricles in his De humani corporis fabrica libri septem (On the fabric of the human body. Book 7: The brain - Vesalius, 1543) before Willis (1664) described the choroid plexus of the fourth ventricle and hypothesized the choroid plexus contained gland-like structures which produced the fluid found in the ventricles. The entire extent of the choroid plexuses was known when Ridley (1695) described the choroid plexus of the third ventricle. After these ancient and distant times, there was a resurgence of descriptions of choroid plexus pathologies in the 1910–1930s (Dandy, 1918; Somerford, 1933; Cohen and Davies, 1939 among others) and more traditional investigative descriptions from the 1960s onwards, first on micrographs about the structure and morphological changes in the shape and distribution of plexus epithelial cells and vasculature (Tennyson and Pappas, 1964; Cancilla et al., 1966; Netsky and Shuangshoti, 1970; Stastný and Rychter, 1976; Sturrock, 1979; Keep and Jones, 1990; Dziegielewska et al., 2001; Liddelow et al., 2010) and later on alterations in the perceived function of the plexus by investigations of cerebrospinal fluid (CSF) (Evans et al., 1974; Johanson et al., 1976; Habgood et al., 1992; Liddelow et al., 2014). Though a wealth of information has been gathered about early developmental origins and growth of the choroid plexus through these means, only recently has research moved to the application of modern technologies to study cues involved in choroid plexus development and maturation. Recent studies on plexus epithelial cell differentiation and genetic lineage analyses along with high throughput genetic expression datasets have provided a wide assortment of data for further investigation. Beyond the historic descriptions of its emergence from neuroectodermal progenitor pools, these recent studies have put emphasis on the importance of the choroid plexuses for normal and regulated development of the remainder of the brain, as well as the role this seemingly small structure plays in normal aging and disease.

In the mouse embryo, the choroid plexus accounts for 27% of the total ventricular wall area (Knudsen, 1964) and in human embryos this value is even larger (approximately 63% of the ventricular surface; Voetmann, 1949), the importance of the choroid plexus in normal brain development and function is therefore immense. This review will spend some time discussing the molecular expression of transcription factors and emergence of the choroid plexuses during early embryogenesis, before embarking on a discussion on disruption of normal plexus mechanisms of action during aging and disease. Due to the excess of information available on the telecephalic (lateral ventricular) choroid plexus, the majority of discussions will be focussed on this plexus, however, when other structures are considered they will be listed as such.

## Normal development

**Figure 1:**
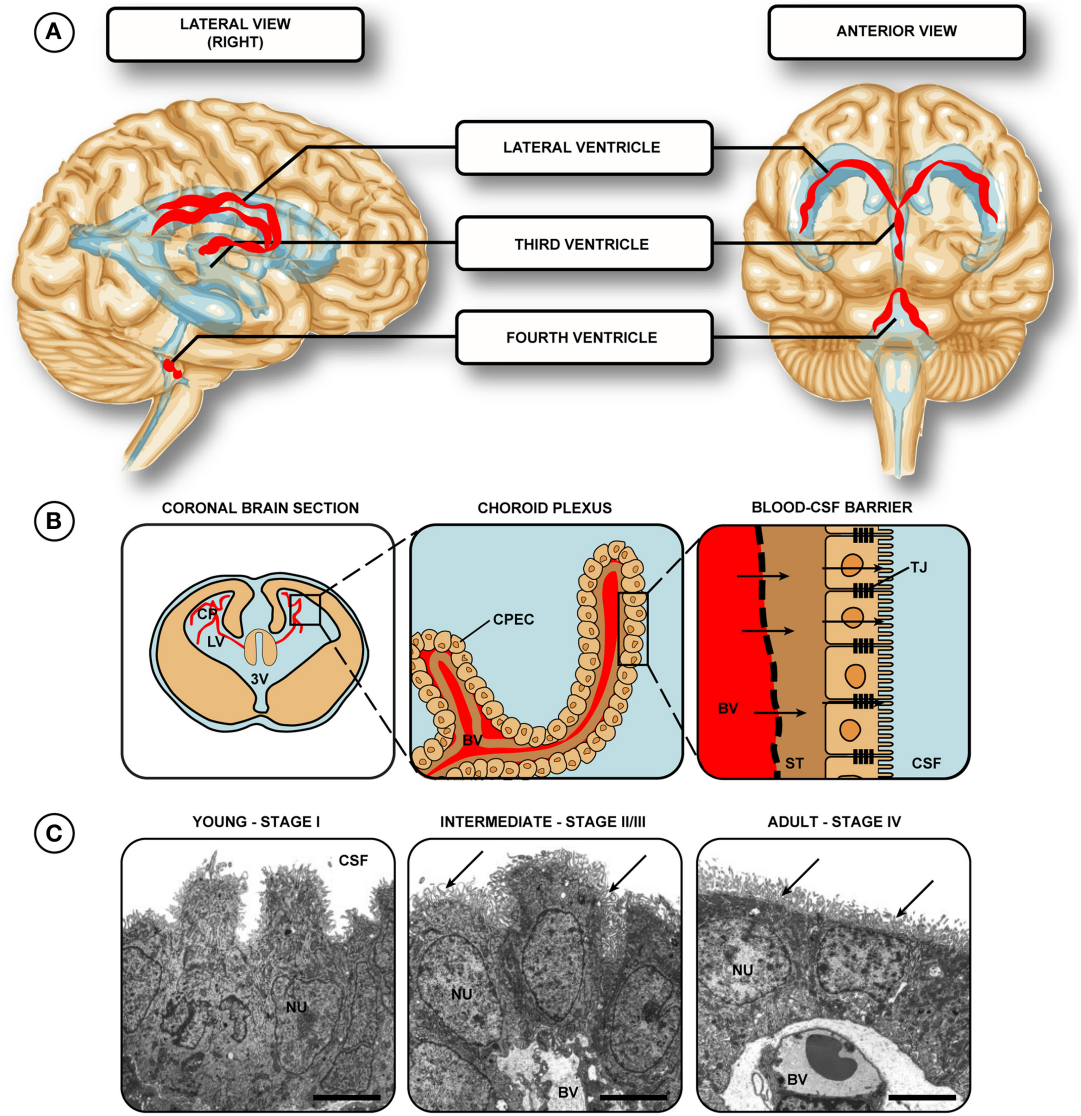
**Location of choroid plexuses in the human brain. (A)** The choroid plexuses are present in the two lateral, third and fourth ventricles (red ribbons). **(B)** Each of the plexuses is comprised of fenestrated vessels, with a single layer of intimately opposed choroid epithelial cells, joined by tight junctions—forming the blood- cerebrospinal fluid barrier. **(C)** Transmission electron micrographs of lateral ventricular choroid plexus. Stage I–the epithelial cells are pseudostratified with centrally located nuclei. Stage II/III–low columnar to cuboidal, basal-to-centrally located nuclei, with apical villi present (arrows, a characteristic from stage III onwards). Stage IV–cuboidal epithelial cells with central-to-apical nuclei and many villi (arrows). **(C)** reproduced from Ek et al. (2003) Copyright © 2003 Wiley All rights reserved. Abbreviations: BV, blood vessel; CP, choroid plexus; CPEC, choroid plexus epithelial cell; CSF, cerebrospinal fluid; LV, lateral ventricle; NU, nucleus; ST, stroma/basement membrane; TJ, tight junction; 3V, third ventricle.

The choroid plexuses, found in the lateral, third and fourth ventricles of the brain (adjacent to the embryonic dorsal midline in the hindbrain, diencephalon, and telencephalon, respectively) are epithelial tissue masses highly vascularized with fenestrated blood vessels (Figure 1). These structures constitute a transfer interface between blood and CSF in the ventricles of the brain. Approximately two thirds of this CSF is produced and secreted by the choroid plexus, the remainder produced by other areas such as the ependymal cells of the ventricular surface and those cells lining the subarachnoid space (see Davson and Segal, 1996; Zheng and Chodobski, 2005 for review). This fluid circulates in the ventricular system, subarachnoid spaces and spinal canal (for review see Davson and Segal, 1996; Saunders et al., 2000; Zheng and Chodobski, 2005) and the plexuses are able to control the homeostasis of its composition by regulation of movement of essential ions and molecules into, and metabolites out of the CSF (Kusuhara and Sugiyama, 2004; Saunders et al., 2013). The choroid plexuses are the main structures that comprise the blood-CSF barrier, the other contributors being the arachnoid and arachnoid villi on the outer surface of the brain (Wright, 1978; and for review see Davson and Segal, 1996). Together these interfaces provide a protective restriction barrier formed by bands of tight junctions between adjacent cells that impede the movement of molecules into the central nervous system (CNS). In addition to this mechanical barrier, the barrier interfaces comprise a biochemical barrier— an additional protective mechanism that effluxes toxic molecules and drugs taken up by cells in the blood-CSF barrier (see Ek et al., 2010; Kratzer et al., 2013). This review will focus on the choroid plexus over the arachnoid membrane, however many properties are retained in both interfaces.

### Gross morphological development

As the choroid plexuses themselves do not have distinct proliferative zones; there is much evidence showing (and is widely accepted) the choroid plexus stroma has mesenchymal origins, like meningeal cells, while the actual plexus epithelial cells are derived from neuroepithelium. Genetic lineage analyses indicate the plexus epithelium originates from the roof plate (Awatramani et al., 2003; Currle et al., 2005; Hunter and Dymecki, 2007) - a well-known signaling center in early CNS development. From this area the migration of pre-plexus cells can be traced. The post differentiated development of the choroid plexus cells themselves has proved a more difficult beast to investigate. Much study using cell division markers have shown no examples of choroid plexus epithelial cells undergoing division to produce additional plexus epithelium, while in contrast, many mitotic figures have been described in the nearby neuroependyma (Zand, 1930; Kappers et al., 1958). An aside should be noted however that there are examples of a proliferative choroid plexus giving rise to neuronal and glial lineage cells (Itokazu et al., 2006; Bolos et al., 2013). The neuroependyma, originally formed by invagination of the anterior end of the neural tube (Imayoshi et al., 2008), proceeds to divide into three regions:

1. the most lateral region gives rise to cortical neuroepithelium, which further develop to neurons and glia of the cortex;
2. the medial or dorsal telencephalic midline regions is split in two, with the cortical hem being a major source of neocortical Cajal–Retzius cells; and
3. the most medial region of the dorsal telencephalic midline gives rise to choroid plexus epithelium.

Systematic studies on histogenesis of plexus epithelial cells report it arises from an infolding of the multilayered roof plate of the neural tube, between the paraphyseal arch and the medial wall (Bailey, 1916; Tennyson and Pappas, 1964; Sturrock, 1979; Thomas and Dziadek, 1993; Awatramani et al., 2003; Currle et al., 2005; Hunter and Dymecki, 2007). In addition, it has been shown with chick-quail chimeras (Wilting and Christ, 1989) that specific cells destined to develop into choroid plexus are detectable up to 72 h before the structure even emerges from the neuroependymal wall of the ventricles. This “pre plexus” empendyma has been shown to envelope connective tissue and develop blood vessels, more representative of a mature plexus, upon direct contact with tissue derived from the mesodermal germ layer (Sarnat, 1998), suggesting cues for plexus differentiation are indeed released by nearby cell types in the developing CNS.

Once entering the plexus, newly differentiated cells undergo maturation through four distinct stages; described in many different species with the distinct difference in marsupials that glycogen is absent (Dziegielewska et al., 2001; Ek et al., 2003). The four stages as described by Tennyson and Pappas (1964) and Sturrock (1979) are as follows: Stage I (see Figure 1C): the early epithelial cells are pseudostratified with nuclei that are centrally located. Stage I plexus epithelia are widely accepted to not contain extensive apical villi (common in the adult plexus). Stage II: low columnar to cuboidal epithelial cell shape, with an emerging basal connective tissue. Apical villi are still absent, though some villi-like extensions are seen in late-stage cells. Nuclei move more apically in the cell. Stage III: now with cuboidal epithelial cells and basally-located nuclei it is in this stage cilia on the apical cell surface appear, along with a great increase in complexity of the capillary network, with an accompanied increase in the number of villi. Stage IV (see Figure 1C): in the final stage of plexus epithelial cell development transition to cuboidal cells is complete and the cells become slightly smaller (approximately 10 μm^2^ in profile), with most nuclei situated centrally-to-basally within the cytoplasm. While additional cells are continuously added to the choroid plexus throughout early development and cell maturation, these newly added cells will transition through the four stages of development outlined. A continued proliferative effort by plexus epithelial cells has been shown to occur even after the original spreading from the neuroependymal wall, though it should be noted that this division usually gives rise to cells that are not reminiscent of plexus epithelium. The choroid plexus epithelial cells from rats from newborns to 8 weeks of age have been shown to function as neural progenitor cells (which can give rise to astrocytes) however this ability decreases with age, with cells from P1 animals twice as likely to undergo this change as those from adult (8 week) animals (Itokazu et al., 2006). It is important to note however this proliferation only occurred after explanting to an ectopic location - continued proliferation of the neuroepedyma which adds additional choroid plexus epithelial cells occurs in adult rat only at an extremely low rate (less than 0.1% of total plexus cells, see Altman and Das, 1965; Doetsch et al., 1999; Liddelow et al., 2010; Johansson et al., 2013). It is interesting to note that in APP/PS1 mice that overexpress amyloid precursor protein and express mutated presilin 1 (important in generation of beta amyloid) there is an almost 5-fold increase in the number of newly divided cells in the choroid plexus at 1 year of age (Bolos et al., 2013). The authors however comment that these newly dividing cells represent proliferation of immature neurons in the choroid plexus and not new plexus epithelial cells *per-se*—suggesting the choroid plexus as an important adult stem cell niche.

### Transcription factors involved in plexus patterning and development

Like all forms of early brain development, growth and maturation of choroid plexus is regulated by coordinated actions of multiple signaling centers at key boundaries between nearby anatomical compartments (refer to Table 1 for a non-complete list). Three neighboring telencephalic midline structures are situated to perform such roles in forebrain patterning: the cortical hem, the septum, and the thalamic eminence at the diencephalic—telencephalic boundary. These structures all express unique complements of signaling molecules. The medial-lateral patterning of dorsal regions of the telencephalon is regulated by a large number of transcription and secreted signaling factors. Some secreted factors, including members of the bone morphogenic protein (BMP) family of proteins, regulate specification of the choroid plexus epithelium by inducing MSX1 and repressing LHX2/FOXG1 levels (Xuan et al., 1995; Furuta et al., 1997; Porter et al., 1997; Monuki et al., 2001; Panchision et al., 2001; Hébert et al., 2002; Bach et al., 2003; Fernandes et al., 2007; Johansson et al., 2013). The cortical hem is a WNT and BMP rich signaling center connected to the choroid plexus on one side and the cortical neuroepithelium on the other (Grove et al., 1998). A lack of the constitutively active form of the receptors for BMPs results in a massive expansion of the choroid plexus epithelium at the expense of the cortical neuroepithelium (Panchision et al., 2001). In contrast, inactivation of BMP receptors results in retarded growth of the choroid plexus, specifically of the epithelial cells (Hébert et al., 2002; Fernandes et al., 2007). One could argue a reduction of BMP receptors on cells in the ventral surface of the neuroepithelium close to the root of the plexus and a large number of these receptors on the dorsal surface may aid in the one-sided growth pattern outlined by Liddelow et al. (2010).

**Table 1:** **Factors involved in the development of the choroid plexus epithelial cells**.

**Gene/Factor**	**Function**	**Species**	**References**
*Bmps*	Misexpression causes expansion of choroid plexus at the expense of cortical neuroepithelium. Deficits cause loss of specification of plexus epithelial cells. BMP4 gives rise to choroid plexus epithelial cells from E10.5 in mouse	Mouse	Panchision et al., 2001; Hébert et al., 2002; Fernandes et al., 2007; Imayoshi et al., 2008
*E(spl)*	Homologs of genes present in mammalian species	Mouse, rat	Kageyama et al., 2007;
*E2F5, foxJ1, p73*	Lack of functional transcription factor causes hydrocephalus due to dysfunction of choroid plexus, mostly dysfunctional cilia on CSF surface of cells and AQP3 channels	Mouse, human (cells)	Chen et al., 1998; Kume et al., 1998; Lindeman et al., 1998; Zheng and Zhao, 2002; Swetloff and Ferretti, 2005; Swetloff et al., 2006
*Hairy, E(spl)*	bHLH repressor genes regulate non-neuronal versus neuronal fate specification in the ectoderm. Up-regulation of *E(spl)* represses neuronal specification, promoting non-neuronal cell fate	*Drosophila*	Campos-Ortega and Jan, 1991
*Hes1, Hes3, Hes5*	Gives rise to choroid plexus epithelial. Knock-out mice have no lateral ventricular choroid plexus	Mouse	Imayoshi et al., 2008
*Lmx1a*	Gives rise to choroid plexus epithelial cells, from E10.5 onwards in mouse	Mouse	Imayoshi et al., 2008
*Msx1/2*	Development of dorsal midline region	Mouse	Hébert et al., 2002; Bach et al., 2003
*Msx1/2*	Induce Fox and Msx genes, causing regulation of specification of choroid plexus epithelium	Mouse	Xuan et al., 1995; Furuta et al., 1997; Porter et al., 1997; Monuki et al., 2001
*Ngn2*	Gives rise to choroid plexus epithelial cells and Cajal-Retzius cells, upregulation causes a loss of choroid plexus epithelial cells	Mouse	Imayoshi et al., 2008
*Notch1*	Expression of activated *Notch1* results in overproduction of hindbrain choroid plexus epithelium (but not of other rhombic lip lineages)	Mouse	Hunter and Dymecki, 2007
*Otx2*	Master regulator of choroid plexus development. Knock-out causes failure of choroid plexus to develop	Mouse	Johansson et al., 2013
*Wnt2b*	Gives rise to choroid plexus epithelial cells, from E10.5 onwards in mouse	Mouse	Imayoshi et al., 2008

**Figure 2:**
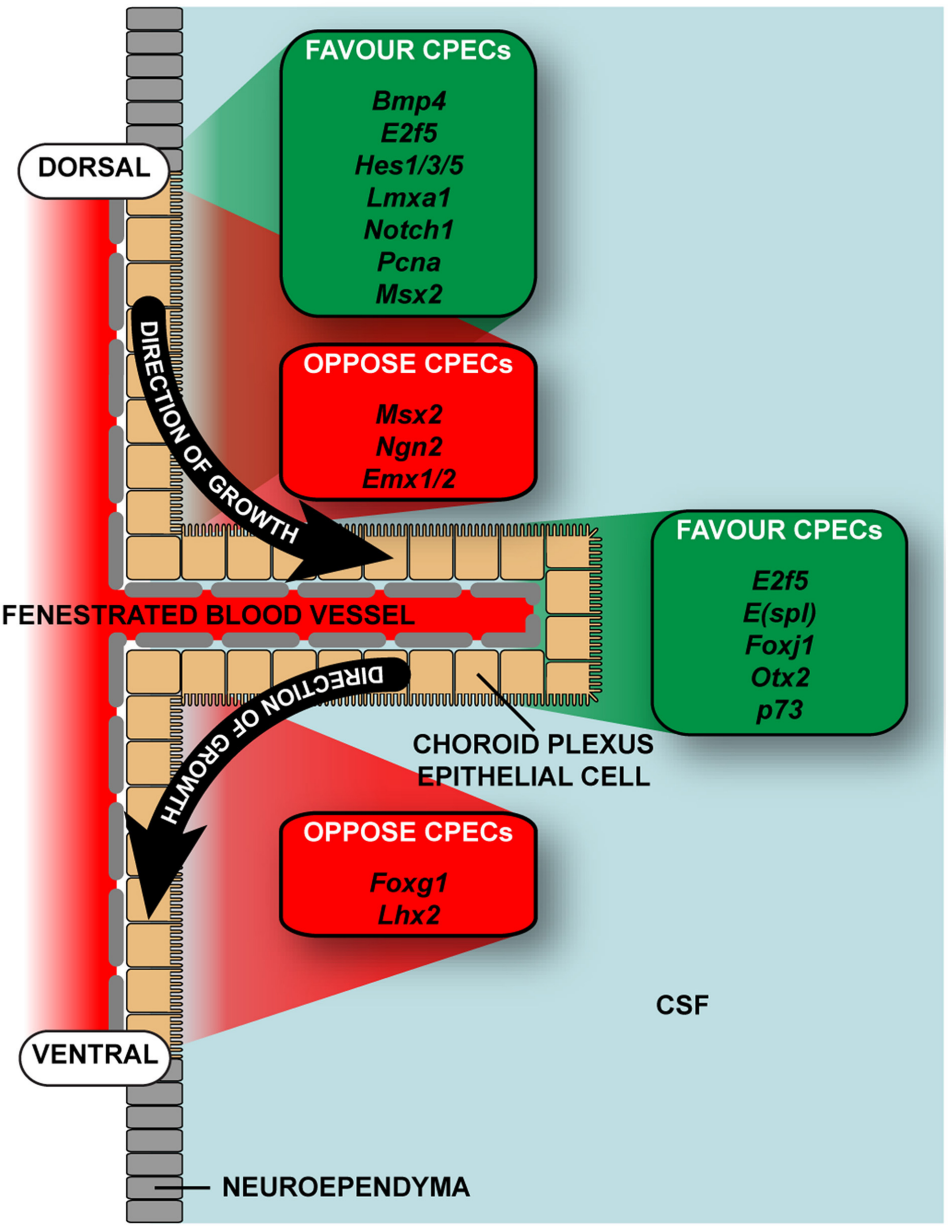
**Location of expression of transcription factors known to be important in plexus development and growth**. The choroid plexus epithelial cells develop from modified neuroepithelium and are only added to the structure from the dorsal surface. No addition of cells is described from the ventral surface, and no transcription factors that promote differentiation into choroid plexus epithelium have been reported as expressed in this region. For full list of transcripts and references refer to Table 1. CSF, cerebrospinal fluid.

*A list of known transcription factors involved in the development of neuroependymal cells of the ventricular wall into choroid plexus epithelial cells. Refer to **Figure 2** for the localization of these factors. This list is by no means exhaustive. Abbreviations: bLHH, basic helix-loop-helix; BMP; bone morphogenic protein; E(spl), enhancer of split; foxJ1, forkhead box J1;Hes, hairy and enhancer of split; Lmx1a, LIM homeobox transcription factor 1α; Ngn2, neurogenin 2; Otx2, orthodenticle homeobox 2; p73, tumor protein 73; Wnt2b, wingless-type MMTV integration site family member 2B*.

The fruit fly *Drosophila* has recently become an invaluable tool in the rapid expansion of the molecular knowledge on the fate specification of cells in the ectoderm. There are several *Drosophila* basic helix-loop-helix repressor genes, such as Hairy/Enhancer of Split [*E(spl)*], able to regulate the non-neuronal versus neuronal specification of cells (Campos-Ortega and Jan, 1991). When cells adopt the neuronal fate they express Delta, which in turn activates Notch signaling in neighboring cells, in turn up-regulating *E(spl)* expression, promoting non-neuronal cell fate (Campos-Ortega and Jan, 1991). This raises the possibility these repressor genes are involved in the formation of non-neuronal tissues in the brain. Mammalian homologs of these genes, the *Hes* gene family, have been shown to be involved in formation of non-neuronal tissue in developing mouse brain (Imayoshi et al., 2008). In these experiments, expression of *Bmp4*, *Hes1*, and *Hes5* is seen in the neuroepithelium directly adjacent to the choroid plexus on the upper (dorsal) surface. This localizes the factors in the correct position to aid in the switch to non-neuronal cell fate required for choroid plexus development. In addition *Hes1* and *Hes5* expression is absent on the lower (ventral) surface of the choroid plexus root, while *Bmp4* expression is still present— possibly causing impedance of mitosis or non-neuronal fate in this area. In addition, inactivation of the repressor genes *Hes1*, *Hes3*, and *Hes5* causes up-regulation of the proneural gene neurogenin-2 (*Ngn2*), prematurely depleting *Bmp* expressing progenitor cells causing loss of choroid plexus epithelial cells (due to the enhanced formation of Cajal-Retzius cells; Imayoshi et al., 2008). A similar effect is seen when bypassing the *Hes* genes and simply up-regulating *Ngn2* expression.

Another set of transcription factors present in choroid plexus epithelial cells immediately after their differentiation from the neuroependyma, *E2f5*, *FoxJ1*, and P73, when expressed in aberrant levels cause non-obstructive hydrocephalus in mouse (Swetloff and Ferretti, 2005). The levels of E2F5 protein in the brain are highest in embryonic development and lower in the adult in mouse (Dagnino et al., 1997; Persengiev et al., 1999; Swetloff and Ferretti, 2005) which correlates well with plexus growth patterns—which is most rapid during early development, but reaches a plateau shortly after birth (Liddelow et al., 2010). The amount of E2F5 protein is also increased in nuclei of choroid plexus epithelium of both mouse and human early in development, suggesting it may be more important for maturation of plexus epithelial cells rather than for the original transition from their neuroependymal cell beginnings (Swetloff and Ferretti, 2005). A summary of the position of these transcription factors is outlined in Table 1 and Figure 2. The proliferative nature of choroid plexus epithelial cells has also been shown to occur even after the original dissemination from the neuroependymal wall and a possible neuronal fate. After a graft of plexus epithelium into the spinal cord of the adult rodent, plexus epithelium is able to differentiate into astrocytes (Ide et al., 2001; Kitada et al., 2001). Though the authors do not comment on what may have caused the switch from epithelial cell to astrocyte, it appears likely some local factor in the environment of the spinal cord has caused the transformation. In addition, choroid plexus epithelial cells from rats at a range of developmental ages from postnatal day 1 to 8 weeks have been shown to have an ability to function as neural progenitor cells, however this ability decreases with age, with the plexus epithelium from P1 animals twice as likely to undergo the change than those from adult (8 week) animals (Itokazu et al., 2006). The considerable potential for growth by the adult choroid plexus is demonstrated by growth of the choroid plexus (almost 40-fold in the adult) by intrathecal infusion of fibroblast growth factor 2 or epidermal growth factor (Itokazu et al., 2006).

More recently, *Otx2* (orthodenticle homeobox 2) has been implicated as the master regulator of choroid plexus differentiation (Johansson et al., 2013). Like *Emx2*, *Otx2* is expressed in the dorsal roof plate and both play important roles in specification of neuroepithelial versus choroid plexus regions (von Frowein et al., 2006). *Emx1* and *Emx2* are regulators able to supress plexus cell fate differentiation, while *Otx2* pushes cells into a plexus epithelial lineage, the two regulators working in tandem to ensure correct pattering early in development. Indeed, deletion of *Otx2* sufficiently early in development causes failure of the choroid plexus to occur, while deletion once the plexus has developed causes epithelial cells to enter an apoptotic pathway, though no apoptotic cells were visualized by the authors (Johansson et al., 2013). These data hypothesize *Otx2* is absolutely required for normal choroid plexus development; however its importance in the general maintenance in the mature/adult choroid plexus is yet to be ascertained. It is known however in humans the obliteration of the lateral ventricular choroid plexus as a treatment for hydrocephalus results in no regeneration of the structure (Hallaert et al., 2012; Ogiwara et al., 2014). This suggests early developmental growth factor patterning cues are paramount for normal plexus development, and the structure cannot regenerate from already-present plexus epithelium.

## Function of choroid plexus epithelial cells

The epithelial cells of the choroid plexus have many functions important for normal embryo development. They are principally recognized as producing a large proportion of the CSF, and acting as site of the blood-CSF barrier, a protective mechanism that ensures the stability of the CSF milieu (Davson and Segal, 1996; Zheng and Chodobski, 2005; Saunders et al., 2008; also see Introduction). Like other brain barriers, the choroid plexus blood-CSF barrier is formed by presence of specialized junctions between adjacent epithelial cells (Wolburg et al., 2001; Saunders et al., 2008; Liebner et al., 2011). The molecular make-up of tight junctions of the blood–CSF barrier is less well-known than those that comprise the blood-brain barrier (Wolburg et al., 2001). A recent study of tight junction protein expression in mouse embryos (E15) and adult choroid plexus has shown several key junctional genes are expressed at a higher level in embryos than in the adults (e.g., *Pcdh*, *Cldn5*), whereas for several other genes the reverse is the case (e.g., *Igsf5*, *Cldn2*, see Liddelow et al., 2012). This is consistent with previous findings that the fundamental functional basis of this barrier, namely occlusion of the paracellular diffusion pathway, is well-established from the earliest stages of differentiation of the choroid plexuses (Bauer et al., 1993; Ek et al., 2003, 2006). The critical function of these junctions is to join the cells together to create a physical barrier to paracellular diffusion, allowing cells to polarize with distinct luminal and abluminal components. The presence of these junctions between cells of the interface between the periphery and the CNS allows cellular transporters to be effective in controlling the distribution of solutes on either side, and thus set up concentration gradients. These gradients are not only important for mature brain function, but are also likely to be significant for essential features of early brain development (cell division, migration, differentiation and synaptogenesis). For instance, the most active transporter in the choroid plexus appears to be the Na^+^/K^+^ ATPase pump—integral for maintenance of the ion gradient that draws water into the ventricles via aquaporin-1 (AQP1) channels in the plexus epithelium (see below). Plexus barrier junctions are tight to molecules as small as lanthanum ion (139 Da; Brightman and Reese, 1969) and tracer molecules such as BED (286 Da; see Ek et al., 2003) and larger dextrans from 30 kDa (Ek et al., 2003, 2006) up to 70 kDa (Liddelow et al., 2009)—more in line with the size of plasma proteins. The presence of this functional barrier is an essential prerequisite for the establishment and maintenance of concentration gradient for ions and proteins between the blood (basal side of the cells) and the CSF on the apical side (see Speake and Brown, 2004). Indeed earlier permeability experiments have already shown that when administered peripherally acidic dyes (Ehrlich, 1885; Goldmann, 1909) or more recently biotinylated and fluorescently-labeled dextran molecules (Ek et al., 2003) only enter the plexus structure and do not enter the CNS milieu. These junctions of the plexus are present from the first emergence of the structure from the wall of the ventricles, however as transcriptome profiling of the possible molecular make-up of this structure has only recently been completed (Marques et al., 2009; Liddelow et al., 2012; Kratzer et al., 2013) there are no plexus-specific knock-out animal models available to further investigate the role of these individual molecules in dysfunctions of plexus development and function.

At all stages of development, the plexus epithelial cells are able to produce and secrete CSF and regulate transfer of molecules across the blood/CSF interface, as tight junctions are present between adjacent cells immediately following their entry from the neuroepithelium. For lipid insoluble substances, such as proteins, this transfer has been shown to be across choroid plexus epithelial cells both in development and in the adult (Møllgård et al., 1979; Ek et al., 2003; Johansson et al., 2006; Liddelow et al., 2009). However, not all choroid plexus cells seem to be involved in this process, the proportion ranges between less than 5% in the adult to about 15% during early stages of brain development in opossum (Liddelow et al., 2009) and rat (Johansson et al., 2006), to over 40% in sheep (Balslev et al., 1997) and humans (Møllgård and Saunders, 1977). These processes of early plexus epithelia will be covered in more detail below.

### Solute carriers

**Figure 3:**
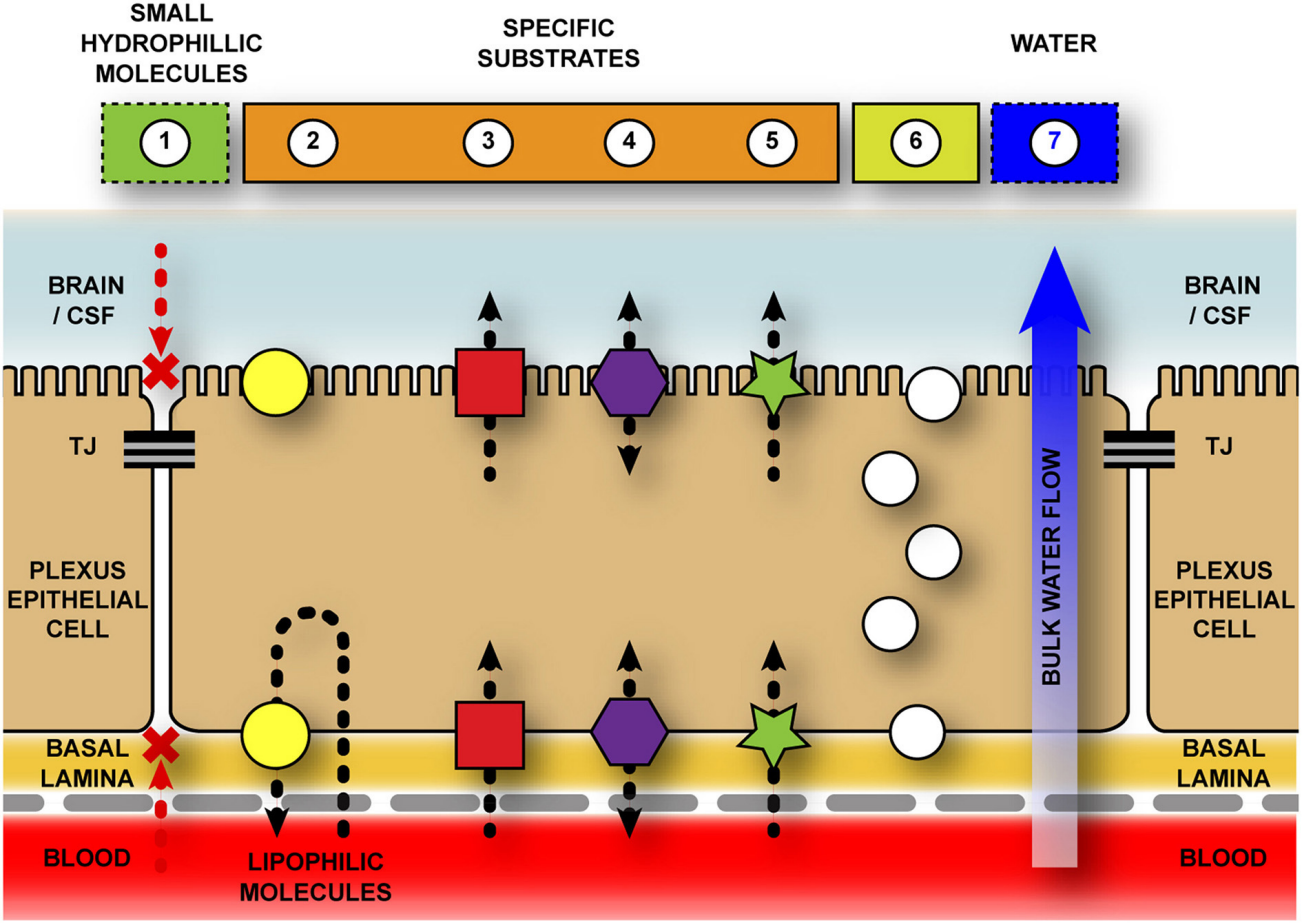
**Schematic representation of transport pathways across the blood-cerebrospinal fluid barrier**. The blood-cerebrospinal fluid (CSF) barrier is formed by tight junctions between neighboring choroid plexus epithelial cells—halting the paracellular movement of molecules both into, and out of, the brain. Additional chemical barriers exist to impede movement of molecules into the central nervous system. 1–Diffusion for hydrophilic molecules through a paracellular pathway is halted by tight junctions (TJ) between adjacent choroid plexus epithelial cells. 2–Efflux transporters (e.g., ABC family) actively remove specific (mostly) lipophilic solutes from cell cytoplasm and extracellular space. Though the majority of evidence suggests a basolateral removal of molecules to the blood space, there is some evidence that ABCB1 (PGP) and ABCG2 (BCRP) localize to the apical membrane of the choroid plexus (Rao et al., 1999; Gao and Meier, 2001; Gazzin et al., 2008; Niehof and Borlak, 2009; Ek et al., 2010; Reichel et al., 2011), however their function in this position is unknown. 3–Inward transporters (e.g., SLC family) actively transport ions, amino acids, and other small molecules across both basolateral and apical surfaces of plexus epithelial cells into the CSF. Without this active transport these molecules would be unable to cross the blood–CSF barriers as they are too hydrophilic and/or highly polarized. 4–Bi-directional transporters (e.g., OAT family). 5–Protein transporters (e.g., SPARC for albumin) specifically target individual proteins (or classes of protein) and transport them across the cells. 6–Vesicular transport/endocytosis due to presence of specific receptors (e.g., transthyretin receptor, TTR; insulin receptor, INSR) or non-specific mediators (e.g., vesicle-associated membrane proteins, VAMPs). 7–Bulk water flow from blood to CSF via aquaporin transporters, specifically AQP1 (Johansson et al., 2005).

Transport across the blood–brain barrier and blood–CSF barrier is directional, with different classes of transporters involved in movement into (e.g., most SLC transporters) and out of (e.g., ABC efflux pumps, see Figure 3). Additionally, there are both active and passive transporters: active mechanisms move solutes up their concentration gradients and require energy (ATP, e.g., Na^+^/K^+^ ATPase), while passive mechanisms move solutes down their concentration gradients and are energy-independent processes. Together, these combined transporters can have a multitude of effects from removal of solutes from the CSF, preventing their entry into the brain (efflux mechanisms), initiation of ion gradients or delivery of specific nutrients, ions and other required molecules to the brain cells (influx mechanisms).

From early in development and throughout adulthood, the occlusion of pathways between plexus epithelial cells produce an environment that allows for gradients to exist across the interface. These ion gradients are due to the presence of transporters on both sides of the choroid plexus epithelium and can transport multiple ions such as Cl^−^/HCO^3−^ on the basolateral surface (Lindsey et al., 1990) or Na^+^/K^+^/Cl^2−^ on the apical surface (Pershing and Johanson, 1982; Plotkin et al., 1997). In addition water channels, namely aquaporin-1, important for water transfer across choroid plexus both in the adult and during development, are found on the epithelial cell surfaces Nielsen et al., 1993; Johansson et al., 2005). Plexus epithelial cells are also able to aid in removal of compounds toxic to the nervous system. In one bizarre case, a person with a bullet wound to the head was reported to have a lateral ventricular choroid plexus engorged with copper and wrapped around the bullet lodged in the ventricle (Buwembo and DeVilliers, 1995)—with Cu^2+^ ion likely being transported out of the ventricles through SLC31A1/SLC31A2 copper transporters on the choroid plexus epithelium (see Saunders et al., 2013).

It has been known for many years that solute carrier (SLC) transporters (for review see Hediger et al., 2004; www.genenames.org/genefamilies/SLC) are both expressed and functionally active in the cerebral endothelial cells that comprise the blood-brain barrier (Kusuhara and Sugiyama, 2004; Simpson et al., 2007; Dahlin et al., 2009; Ho et al., 2012). It is now known that over 65% of the nearly 400 SLC transporters are also expressed by epithelial cells of the choroid plexus (Saunders et al., 2013). The main transporters defined from physiological studies are those for glucose (GLUT-1), amino acids (acidic, basic, neutral) monocarboxylic acids and ions, including metabolically important ions such as Fe^2+^, Cu^2+^ and Mg^+^ (Bito and Myers, 1970; Bradbury et al., 1972; Amtorp and Sørensen, 1974; Oldendorf, 1977; Cornford et al., 1982; Morgan and Moos, 2002; amongst others). As there is such a large number of SLC transcripts, it can prove difficult to assign these examples of more specific molecules for which there is already a wealth of physiological evidence of transport available. It was assumed in many early studies focussed on entry of molecules into brain that entry was via the blood–brain barrier interface (e.g., Oldendorf, 1977; Cornford et al., 1982; Lefauconnier and Trouvé, 1983) and the extent of transport of a second entry route possible via the choroid plexuses was not fully investigated. In addition, it was not clear from some of these reports if the CSF and choroid plexuses had been removed prior to analysis of brain samples - meaning any choroid plexus tissue or CSF included in brain samples may have led to an overestimate of the contribution of blood–brain barrier transport of the amino acids into the brain. This contamination though small is possible to alter the outcome of experiments as at least some amino acids are reported to accumulate in the choroid plexuses (al-Sarraf et al., 1997a) in addition to entering the CSF directly. Separate assessment of CSF in the developing brain, mainly reflecting entry across choroid plexuses, has been examined by Segal and colleagues (al-Sarraf et al., 1997b). More recently, expression of a large number of SLC transporters has been reported in the embryonic and adult choroid plexus (Marques et al., 2009; Liddelow et al., 2010, 2012; Ho et al., 2012; Saunders et al., 2013), however little physiological investigation has been completed on their function or activity.

Aquaporin-1, the main water channel in the choroid plexus, is present in plexus epithelial cells as soon as they begin differentiation (Johansson et al., 2005), making water transport possible from the earliest moments in choroid plexus development. The gradient that drives the water transport however does not appear to be regulated in development in the same manner as in the adult (Johansson et al., 2008a; Liddelow et al., 2013). The two components that have proved crucial for driving an influx of water and thus production of CSF: Na+/K+ ATPase and carbonic anhydrase (for review see Davson and Segal, 1996; Catala, 1998; Zheng and Chodobski, 2005), are not present in the early developing choroid plexus (Johansson et al., 2008a). An early function water influx may be due to the high protein concentration in the CSF of these young animals - causing an osmotic pressure gradient across the choroid plexus (see below and Johansson et al., 2008b).

The ever complex mammalian brain contains diverse cell types and distinct microstructures bound and protected by a number of both physical and enzymatic/physiological brain barriers. The large numbers of transporters present at the blood–CSF barrier likely play crucial roles in energy metabolism, nutrient supply, as well as CSF production and neurotransmitter regulation in the brain, particularly during development. The now debunked dogma that the brain develops without functional brain barriers has been replaced with the realization that dysfunction of specific transporter mechanisms, either genetic or acquired through some pathological process, likely play more important roles in subsequent mal-development of the brain or neuropsychiatric disorder later in life than. Thus these transporters are also potential pharmaceutical targets for treatment of such diseases, as they are possible targets for drug entry and toxin removal in both the developing and adult brain.

### Protein

CSF in the developing brain contains characteristically high concentrations of plasma-derived protein when compared to the adult. Levels for embryonic mice and rats are in the range of several 100 mg/100 ml, whereas in the adult these levels fall to around 10 mg/100 ml (Davson and Segal, 1996), while over the same time the level of plasma proteins circulating in the blood increases from several 100 mg/100 ml to several thousand. The same trends are consistent for rodents, marsupials and humans. The main route of entry for this protein from plasma into CSF and brain is via specific protein-transferring cells of the choroid plexus (Møllgård and Saunders, 1977; Dziegielewska et al., 1980; Johansson et al., 2006; Liddelow et al., 2009). Although the route of this transfer has been identified as intracellular, the actual mechanism remains unknown (Knott et al., 1997; Liddelow et al., 2009). Once proteins have transferred across the choroid plexus into CSF, some are taken up into cells in the brain, while others continue through the ventricular system to be reabsorbed by the arachnoid granulations on the outer surface of the brain. For example some neuroependymal cells lining the cerebral ventricles take up proteins such as albumin and the fetal protein fetuin (Dziegielewska et al., 2000). The initial cells that form the first layers of the neocortex in the embryo take up fetuin via apical dendrites in contact with the dorsal surface of the cortex (Dziegielewska et al., 2000). Other plasma proteins such as albumin and alfa-fetoprotein are also transported via the same route from early during development, however, this uptake has been little studied and it is not clear whether the proteins themselves are functionally important or bound ligands such as hormones and growth factors are the precious cargo. A recent publication indicated the number of plasma protein positive cells in the ventricular zone of a fetal mouse can be increased following an inflammatory response of the dam—indicating protein uptake by the brain can be physiologically responsive to alterations in uterine environment (Stolp et al., 2011). It is also known that this choroid plexus protein transport system is dynamic—able to adapt to acute alterations in levels of individual proteins circulating in the plasma (Liddelow et al., 2014). What is not known is whether or not the choroid plexus is able to maintain the protein composition of the CSF after chronic alterations in circulating proteins.

The first cells that differentiate to become choroid plexus epithelial cells appear able to transport protein immediately, with no apparent preference provided to cells of different stages of development. Although there appears to be a higher degree of specificity for individual proteins earlier in the development (Liddelow et al., 2010), adult cells are equally able to bulk transfer a range of proteins into the CSF without there being any trauma or damage to the blood-CSF barrier interface. The requirement for protein in the CSF and CNS is likely two-fold: initially the high protein concentration reported in the CSF of early developing animals sets up an osmotic pressure gradient causing the influx of water and consequently improving ventricular expansion and normal brain growth and development (Johansson et al., 2005); and secondly, these plasma proteins are constantly attached with large number of growth factors and other required molecules for normal CNS maintenance—thus active transport of protein into the CSF is a way to control import of other factors not produced locally (Liddelow et al., 2012).

## Aging and disease

**Figure 4:**
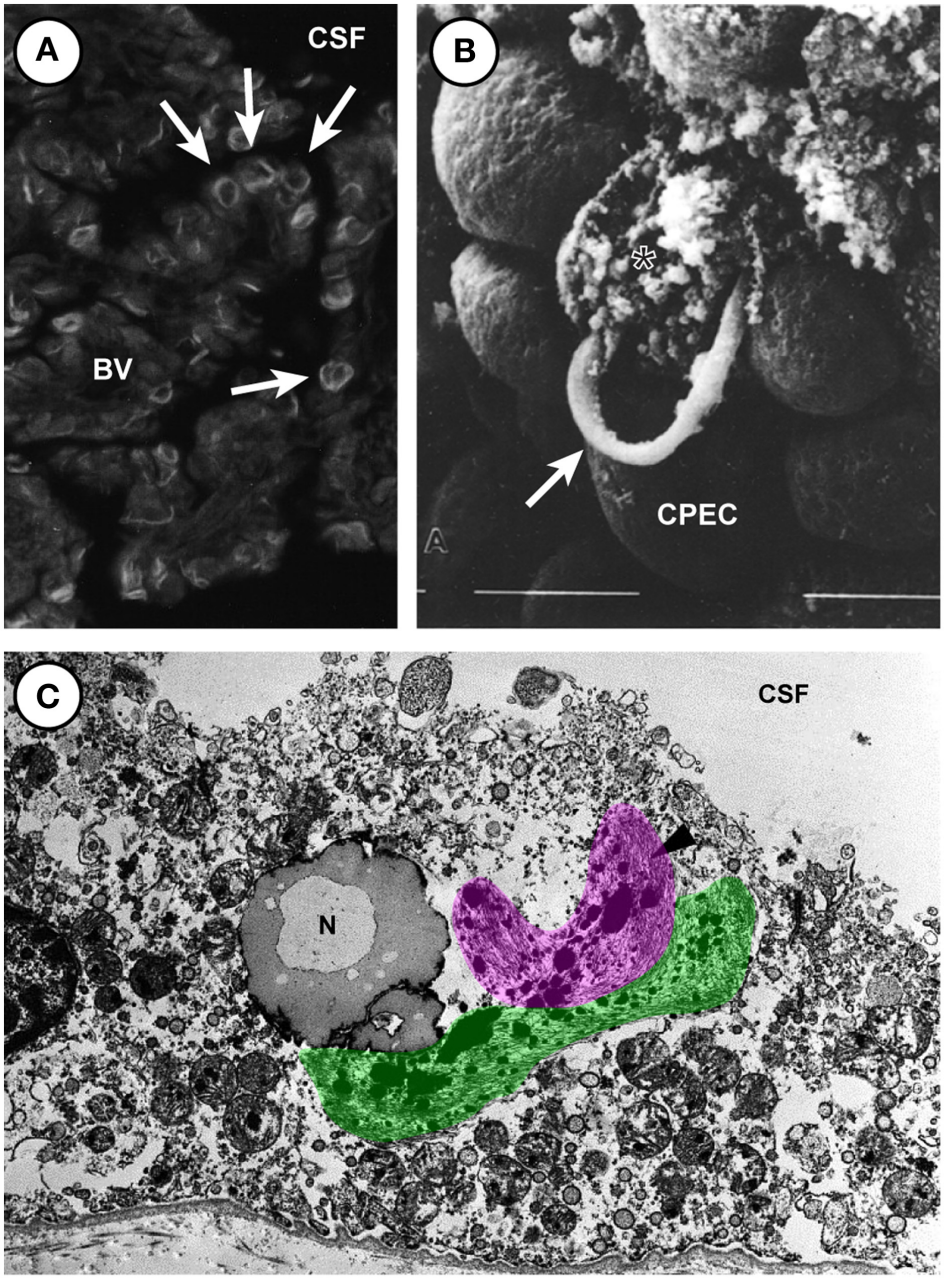
**Biondi ring tangles in aged human choroid plexus of the lateral and fourth choroid plexus. (A)** Fluorescent light micrograph of thioflavin S-stained Biondi ring tangles (arrows) in the choroid plexus of the brain of a 78-year-old human female with Alzheimer's disease showing Biondi ring tangles appear as ring, tangle, serpentine, and curled profiles. (Magnification: 530×). **(B)** Scanning electron micrograph (Magnification 2500 ×) showing destruction of plexus epithelial cell containing ring-like Biondi inclusions. Arrow marks a ring bursting from an individual plexus epithelial cell. Material from 78 year old woman. **(C)** Electron micrograph of a choroid plexus from a 70-year-old male with Alzheimer's disease showing the fibrous Biondi ring tangles (one highlighted in pink, the other in green) associated with lipofuscin granules, mitochondria, and other cellular components. (Magnification: 10,300×). **(A,C)** Reproduced from Wen et al., 1999 Copyright © 1999 Elsevier Science B.V. All rights reserved. **(B)** Reproduced from (Kiktenko, 1986) Copyright © 1986 Springer All rights reserved. Abbreviations: BV, blood vessel; CPEC, choroid plexus epithelial cell; CSF, cerebrospinal fluid; N, nucleus.

The choroid plexus is unique in the CNS in that the cells, once born and fully matured, do not undergo replacement or degeneration under normal conditions. In the adult, the proliferation of choroid plexus epithelium has been shown to occur at a low rate—less than 0.1% of total plexus cells per day (Altman and Das, 1965; Doetsch et al., 1999; Liddelow et al., 2010), but no data are available on the senescent plexus. There are few reports of disease of the choroid plexus epithelium itself many cases of plexus disability result from endothelioma of the blood vessels, invading metastatic cancerous growth (accounting for less than 0.14% of all cerebral metastases—Gopal et al., 2008), or inflammatory lesions, however papilloma of the epithelium itself seems to be the most common, though still only accounting for less than 1% of all brain tumors (see Rickert and Paulus, 2000 for review). It is important to note however the high expression of Vimentin *(Vim*) by nearly all choroid plexus tumors suggests a vascular cell abnormality, while the presence of the astrocytic marker glial fibrilliary acidic protein (*Gfap*) in up to 50% of plexus neoplasms suggests some conversion of epithelial cells to glial lineages (Rickert and Paulus, 2000), as has been reported for plexus implantation in spinal cord (Ide et al., 2001; Kitada et al., 2001). Other reports in multiple sclerosis describe HLA-DR (a MHC class II cell surface receptor encoded by the human leukocyte antigen) composites on choroid plexus epithelial cells (Vercellino et al., 2008), however closer inspection of the micrographs suggest these deposits are actually present in epiplexus cells as they are never central to the epithelial cell and always contain a separate nucleus. It seems that regardless of case-study age, race, or location, these diseases of the choroid plexus are rare, and to date no study descriptions or animal models are able to accurately describe insufficiencies in normal plexus function (although anencephalic expansion due to excess CSF production by the choroid plexuses is widely reported). Rarer still are descriptions of changes in the choroid plexus during normal aging. The choroid plexus performs continuous and vital function, producing up to 75–90% (approximately 450–1000 ml/day) of the CSF (see Davson and Segal, 1996). This fluid adequately nourishes the brain prior to full vascularization (see above) and provides buoyant suspension and protection to the brain and spinal cord. During normal aging there have been reports of filamentous, ring-like or arc-like structures in the epithelium of the choroid plexus, termed Biondi bodies (Biondi, 1933; Oksche and Kirschstein, 1972; Kiktenko, 1986; Wen et al., 1999; see also Figure 4). Structurally these filamentous rings are associated with lipid droplets, and appear to develop within the epithelial cells themselves (and thus may be agents of cellular destruction) rather than in the extracellular matrix, however the study by Kiktenko (1986) makes special mention of the fact they were unable to find convincing signs of damage in plexus epithelial cells with large-sized Biondi bodies. Inspection of the electron micrographs however clearly shows examples of these tangled rings bursting forth from ruptured plexus epithelial cells, while nearby ring-free cells are noticeably undamaged (see Figure 4B). These Biondi bodies, or Biondi ring tangles, are not seen between adjacent cells - only in the cytoplasm of plexus epithelia. This intracellular location of the Biondi bodies and their state of preservation compared to other cytoplasmic elements suggest a destructive effect on epithelial cells of choroid plexuses. The more common occurrence in Alzheimer's disease (Miklossy et al., 1998) suggests dementia disease states may cause premature aging of the choroid plexus, as epithelial atrophy is significantly accentuated (as reported by a decrease in cell height is observed compared to age-matched controls (Serot et al., 2000). Choroid epithelial cells also acquire numerous other lipofuscin vacuoles (along with Biondi bodies) in Alzheimer's patients (Miklossy et al., 1998; Wen et al., 1999; Alvira-Botero and Carro, 2010). These Biondi inclusions have thus far proved difficult to image extensively, as the presence only in higher-order primates and humans presents issues for both tissue availability and preservation. The light and transmission electron micrographs provided by Wen et al. (1999; see Figures 4A,C) come from material obtained from tissue banks and as such no information is provided on tissue preparation protocols. Older material prepared for scanning electron microscopy by Kiktenko (1986; see Figure 4B) was prepared from a wide range of aged and otherwise healthy human autopsies that were fixed within 1.5–4.5 h after death. In these images it is possible to see choroid plexus epithelial cells bursting, possibly due to Biondi ring inclusions and not an artifact of fixation as all burst cells contain these ring structures. Whatever their effect on the plexus, it is true to say that Biondi bodies are characteristic of choroidal epithelia of aged humans. Their absence in young-to-middle aged non-human primates, as well as their absence in various senescent mammals (rodents, dogs, and cats) and birds, has led to suggestions they may relate to differences in brain senescence between humans and other animals. However, Biondi-like inclusions have been identified in an aged (43 year old) male chimpanzee (Oksche et al., 1984).

The choroid plexus therefore demonstrates a robust accumulation of pathological changes, in the form of Biondi bodies, with normal aging, however the changes to normal function are difficult to ascertain, as no rodent examples are reported thus study of their effects cannot be completed. It is likely however these modifications could alter choroid plexus function, including synthesis, secretion, and transport of proteins and other molecules. A possible reason for this amazing cellular longevity is the high expression of the aging repressor Klotho (*Kl*, see Liddelow et al., 2012). KLOTHO protein is a serum circulating factor that declines with age (Kuro-o et al., 1997; Xiao et al., 2004). Mice deficient for KLOTHO protein manifest a syndrome similar to accelerated human aging and display rapid and extensive arteriosclerosis. Although the vast majority of research has been based on lack of KLOTHO, it is demonstrated an overexpression of *Kl* transcript in mice extends their average life span between 19 and 31% compared to normal mice (Kurosu et al., 2005). The high expression of *Kl* throughout plexus development and into the adult suggests some protective effect on plexus epithelial cells themselves, and also on other CNS cells— especially considering evidence that KLOTHO protein levels in the CSF are decreased in Alzheimer's disease humans (Semba et al., 2014).

To date, the only study of the choroid plexus in systemic disease has been reported by Dohrmann and Herdson (1969). They used NZB/NZW hybrid mice, a strain which develops a disease resembling human systemic lupus erythematosus. Fine structural examination of the choroid plexus revealed an irregular, homogeneous thickening of the capillary basement membranes. Other miscellaneous reports on the pathology of the choroid plexus include Hoff (1930), Rand and Courville (1931) and Leviton et al. (1977). Hoff (1930) reported on increased permeability of the choroid plexus in experimental head injury. Another survey of 62 cases of fatal cerebral trauma noted oedema of the stroma and vacuolation of the epithelial cells of the choroid plexus (Rand and Courville, 1931). The presence of amyloid in the choroid plexus of elderly brains was described by Divry (in Blackwood et al., 1963). Amyloid was present within the choroidal epithelium, confined to the free margins of the cells.

## Concluding remarks

On February 7th 1922, Sir John Bland-Sutton gave what was by all accounts an energetic emeritus lecture at Middlesex Hospital, Enland, entitled “The choroid plexus and psammomas.” As an introduction to the choroid plexus he commented “The ruffle-like structures, called choroid plexuses, which float in the ventricular fluid of man's brain, have aroused the curiosity of anatomists for centuries, yet they took little more interest in them than gardeners take in weeds.” Though we have moved on from humble gardeners, until recently the neuroscience community has lagged in the uptake of important studies focussed on the choroid plexus. As a structure that produces and secretes CSF, controls and protects the internal environment of the adult CNS, and is present even before vascularization of other cortical structures, surely we have sufficient evidence to justify serious investigations into this small epithelial tissue mass in the ventricles of all vertebrates. This review has touched on the early development of the choroid plexuses, and its emergence as a possible location of serious complications in aging and disease. Indeed the close homeostatic control of the internal milieu of the CNS: the CSF, glia, and neurons, plays a vital role in normal and abnormal brain function. Dysfunctions of all of the brain barriers contribute heavily to the pathology of neurological conditions, however the added detriment of a dysfunctional choroid plexus during development is additional reason for concern. A proper understanding of the choroid plexus will likely prove important for the production of drug delivery and therapies to help ameliorate a wide range of neurological diseases.

### Conflict of interest statement

The author declares that the research was conducted in the absence of any commercial or financial relationships that could be construed as a potential conflict of interest.

## Abbreviations

CNS: central nervous system
CSF: cerebrospinal fluid
SLC: solute carrier
